# Mr. Ring, on Vaccination

**Published:** 1803-01-01

**Authors:** John Ring

**Affiliations:** New Street, Hanover Square


### *To the Editors of the Medical and Phyfical Journal.*

Gentlemen,

I Beg leave to observe, with respect to the last letter of Mr. Pears, that I have never questioned the fidelity of his report; but the evidence of the different persons, which he has thus faithfully reported, is at variance with itself, and with truth; and can be of very little service to any cause.

Happily, however, Vaccine Inoculation requires no such aid. It is now patronized by several crowned heads. The King of Prussia, who long since adopted it in his own family, has now established an Institution for that purpose at Berlin. The Empress Dowager of Russia has also honoured the practice with her peculiar protection. The Emperor of Germany has submitted two of his children to this salutary operation; and the King of Sweden has not only caused his infant daughter to be vaccinated, but also established a Vaccine Institution at Stockholm.

Most

Most of the principal cities of Europe have erected institutions of this kind; and the City of London, if last, will not, I trust, be least in following the glorious example.

The case mentioned by Dr. De Carro is not the earliest instance of Vaccination. On the 6th of August, 1800, I inoculated a child of Mr. Austin, who now lives in Globe Street, Bethnal Green, immediately after his birth, to protect it from the small-pox, under which another child in the same family then laboured. The operation, in all respects, proved successful; and the child has since that time been often exposed to the small-pox with impunity.

I have ascertained, that the cow-pox has existed in Hertfordshire; a county in which we have no record, as far as I can recollect, of its having been noticed on any other occasion.

I am informed by Dr. Crichton, that some vaccine matter, with which I supplied him, has proved successful in South America. It was sent to Demarara, by Mr. Skerrett of Ragmore House in Hampshire, who communicates to Dr. Crichton the agreeable intelligence, that it has been tried with success in the garrison, almost entirely consisting of black people, and in several plantations in the colonies of Demarara and Essequibo; and that it was becoming general in those places. The appearance of the disease was the same as in Europe.

Dr. Waterhouse, of Cambridge, New England, inoculated six persons with two toothpicks, well charged with vaccine matter, which he had received from me, without diluting the matter; and succeeded in every case. He also succeeded with a thread ninety days old, without moistening the matter.

When inoculation is performed with a toothpick, a puncture is first to be made with a lancet; then the point of the toothpick is to be inserted into the puncture, and to be held there some time. Afterwards, the flat sides of the toothpick are to be drawn repeatedly over the puncture, in order to fill it as much as possible with the matter. Previous dilution is not only unnecessary, but is supposed to be one of the most common causes of failure.

Dr. W. informs me, that the first matter with which he succeeded, was not from the Vaccine Pock Institution, as Dr. Pearson supposes; but from the stock of Dr. Jenner, through the hands of Mr. Creaser and Dr. Haygarth. It was preserved on a thread, corked up in a common vial. This he wishes to be published. I am, &c.

*New Street, Hanover Square, Dec. 20, 1802.* JOHN RING.

